# Features and In Vitro Assessment of Antiviral Activity of Organic Coatings Doped with Silver-Based Compounds Against Human Coronavirus

**DOI:** 10.3390/ijms262211068

**Published:** 2025-11-15

**Authors:** Maja A. Zaczek-Moczydłowska, Bartosz Kopyciński, Alicja Hryniszyn, Małgorzata Osadnik, Anna Czech, Krzysztof Pęcak, Aleksandra Markowska, Saeid Ghavami, Krzysztof Matus, Ewa Langer, Marek J. Łos

**Affiliations:** 1 Biotechnology Center, The Silesian University of Technology, 44-100 Gliwice, Poland; 2 Doctoral School, The Silesian University of Technology, 44-100 Gliwice, Poland; 3 Łukasiewicz Research Network, Institute of Polymer Materials, 87-100 Toruń, Poland; 4 Łukasiewicz Research Network, Institute of Non-Ferrous Metals, 44-100 Gliwice, Poland; 5 Faculty of Medicine, Medical University of Warsaw, 02-091 Warsaw, Poland; 6 Department of Human Anatomy and Cell Science, Rady Faculty of Health Sciences, Max Rady College of Medicine, University of Manitoba, Winnipeg, MB R3E 0T6, Canada; 7 Faculty of Medicine, Academy of Silesia, 40-555 Katowice, Poland; 8 Paul Albrechtsen Research Institute, Cancer Care Manitoba, Winnipeg, MB R3E 0V9, Canada; 9 Faculty of Mechanical Engineering and Technology, The Silesian University of Technology, 44-100 Gliwice, Poland

**Keywords:** human coronavirus, SARS-CoV-2, biomaterials, antiviral coatings, AgNP

## Abstract

Implementation of novel antiviral coatings and textiles, which can be utilised in the production of personal protective equipment, has the potential to enhance public health security against future pandemic outbreaks. Respiratory viruses, particularly SARS-CoV-2, responsible for COVID-19, have emerged as a major global concern due to their rapid transmission and high mortality rates, leading to nearly seven million deaths worldwide between 2020 and 2025. This statistic underscores the necessity for the development and implementation of advanced antiviral materials to prevent viral infections. This research focused on the in vitro evaluation of the antiviral properties of three antibacterial compounds containing silver (Ag) that were functionalized with coatings. We assessed onsite synthesised Ag powder in comparison to commercially available antibacterial additives, which included nanosilver on colloidal silica (AgSiO_2_) and silver sodium hydrogen zirconium phosphate (AgNaOPZr), as potential antiviral agents in coatings against human coronavirus (HCoV). Antiviral assessments revealed that coatings containing Ag at higher concentrations (2.5 and 5%) exhibited limited antiviral effectiveness, with a titer reduction in log < 2. In contrast, the functionalization of AgSiO_2_ on coatings significantly suppressed viral replication resulting in a notable reduction in virus titer of log ≥ 2 for all tested concentrations.

## 1. Introduction

Coronaviruses that infect humans belong to a group of enveloped, single-stranded RNA viruses classified under the Coronaviridae family and the Nidovirales order [1]. This family comprises seven strains known to cause diseases in humans, which include four HCoVs: OC43, NL63, 229E, and HKU1, along with Severe Acute Respiratory Syndrome Coronavirus (SARS-CoV), Middle East Respiratory Syndrome Coronavirus (MERS-CoV), and Severe Acute Respiratory Syndrome Coronavirus-2 (SARS-CoV-2). These viruses can lead to respiratory illnesses that range in severity from mild colds to severe conditions such as pneumonia, acute respiratory syndrome, and chronic diseases [2]. The COVID-19 pandemic, instigated by the rise in SARS-CoV-2, has exerted a worldwide influence, with more than 777 million cases reported and around seven million fatalities anticipated by 2025, reported to the World Health Organisation [3].

The worldwide health emergency caused by SARS-CoV-2 has initiated a surge of investigations into viral genomics [4], advancements in vaccine technology [5], and the evaluation of various antiviral substances, which encompass metallic elements (e.g., Ag, Au, Cu, Si), oxides (e.g., TiO_2_, CuO, FeO), fullerenes, nanotubes, polymers, engineered nanoparticles, and composites to prevent disease spread [6,7,8,9]. In recent years, Ag nanoparticles (NPs) have exhibited promising antiviral properties against a range of viruses, such as influenza A (H1N1), HIV-1, and SARS-CoV-2, by interfering with viral entry, replication, or structural integrity [10,11,12]. AgNPs have attracted considerable attention due to their well-established antiviral effectiveness against SARS-CoV-2, coupled with low toxicity, as evidenced by in vitro studies [10,13,14,15], in silico analyses [15,16], and in vivo applications [13]. The recognised potential mechanisms through which AgNPs exert their antiviral effects include binding to viral surfaces, generating reactive oxygen species (ROS) that compromise viral structure, enhancing the host’s immune responses, and inhibiting viral replication and spread [17,18].

The antiviral efficacy of AgNPs is notably affected by their dimensions, shape and surface charge. In a particular investigation, polyvinylpyrrolidone-coated approximately 10 nm AgNPs demonstrated a reduction in SARS-CoV-2 infectivity at concentrations ranging from 1 to 10 ppm, while cytotoxic effects were observed at around 20 ppm and higher [10]. Furthermore, the effectiveness of AgNPs was found to be significantly influenced by both particle size and surface coating: for instance, 50 nm branched polyethylenimine-coated AgNPs displayed the most pronounced antiviral effect among ten different types evaluated. The surface chemistry and potential difference in coated AgNPs show a positive correlation with antiviral potency (R^2^ ≈ 0.82) [19]. Coatings such as chitosan on AgNPs further improve safety and selectivity—chitosan-AgNP composites inhibited ACE2/spike binding (IC_50_ ≈ 303 µg/mL), RdRp (IC_50_ ≈ 32.7 µg/mL), and CTSL (IC_50_ ≈ 13.8 µg/mL) while simultaneously decreasing tissue toxicity in vivo [20]. Recent research indicates that incorporating silver nanoparticles into inert support matrices like silica (SiO_2_) or zirconia (ZrO_2_) can markedly improve antiviral efficacy by enhancing dispersion, minimising aggregation, and regulating Ag ion release. For instance, Assis et al. illustrated that a SiO_2_–Ag composite embedded in an ethyl–vinyl–acetate matrix achieved swift inactivation of SARS-CoV-2, underscoring how SiO_2_ supports preserve the high surface activity of Ag while mitigating the loss of active surface area [21]. Although there is a scarcity of specific antiviral studies on Ag–ZrO_2_ systems, a recent study revealed that a SiO_2_–ZrO_2_ hybrid matrix containing Ag NPs retained nanoparticle stability for over 500 days in ambient conditions, indicating enhanced durability that could be advantageous for antiviral surface coatings [22]. Therefore, matrix support (SiO_2_ or ZrO_2_) not only physically stabilises AgNP but also influences their interaction kinetics with viral particles—likely by facilitating closer contact with virions and sustained ion diffusion instead of burst release [22].

Research has shown that AgNP coatings on surgical masks can achieve up to 100% inactivation of SARS-CoV-2 after 60 min of contact, with notable viral reduction occurring even after just 1 min [23]. Ag-based coatings, including those that utilise SiO_2_, have been effectively employed on protective face masks to improve their antiviral performance. Notably, AgNPs incorporated into coatings or composite materials—such as Ag-nanocluster/silica coatings on polypropylene masks—achieved nearly complete inactivation of SARS-CoV-2 after one hour of contact [24].

Nevertheless, the increasing utilisation of AgNPs has prompted concerns regarding their potential cytotoxic effects on human cells. Recent investigations indicate that AgNPs can trigger oxidative stress, damage to mitochondria and lysosomes, and apoptosis in different human cell types, such as endothelial cells and hepatocytes [25,26]. The degree of cytotoxicity is affected by several factors, including particle size, shape, surface charge, dosage, duration of exposure, and the type of cell involved [27,28]. For instance, AgNPs smaller than 10 nm can more easily penetrate cell membranes and may produce elevated levels of reactive oxygen species (ROS), resulting in increased toxicity [28].

The aim of this study was to assess the antiviral properties and cytotoxicity of developed coatings that were functionalized with Ag, AgSiO_2_, and AgNaOPZr in vitro. The characteristics of these functionalized bioactive materials on coatings were analysed using SEM or/and S/TEM, elemental EDS/EDX techniques. To determine antiviral activity against SARS-CoV-2, we utilised the model virus HCoV-229E, which allowed us to conduct tests under Biosafety Level 2 conditions.

## 2. Results

### 2.1. Characteristics of Bioactive Substances and Coatings

Three bioactive substances have been incorporated into green (Gr) organic coatings at various concentrations (5%, 2.5%, 1.25%, 0.625% and 0%), and prepared for antiviral tests as discs with a diameter of 20 mm and a thickness of approx. 85 µm.

The characteristics of three bioactive compounds used for the functionalization of Gr organic coatings are presented in Table 1.

**Figure 1:**
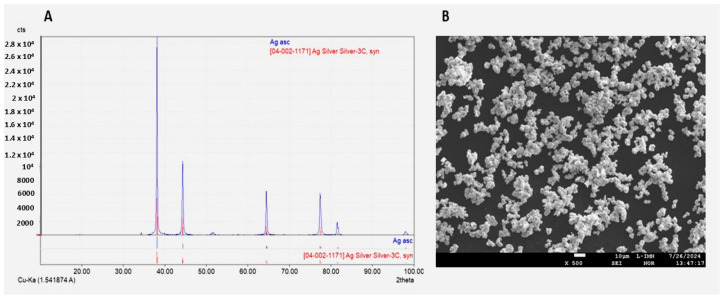
X-Ray Diffraction (XRD) of Ag powder (**A**); EPMA image of synthesised Ag particles (**B**).

Results obtained from the diffractogram of synthesised Ag powder assigned Ag elements (Figure 1A). Particle size distribution results indicated the average particle size approx. up to 3 μm (Table S1). Electron probe microanalyzer (EPMA) indicated agglomerated Ag particles (Figure 1B).

**Figure 2:**
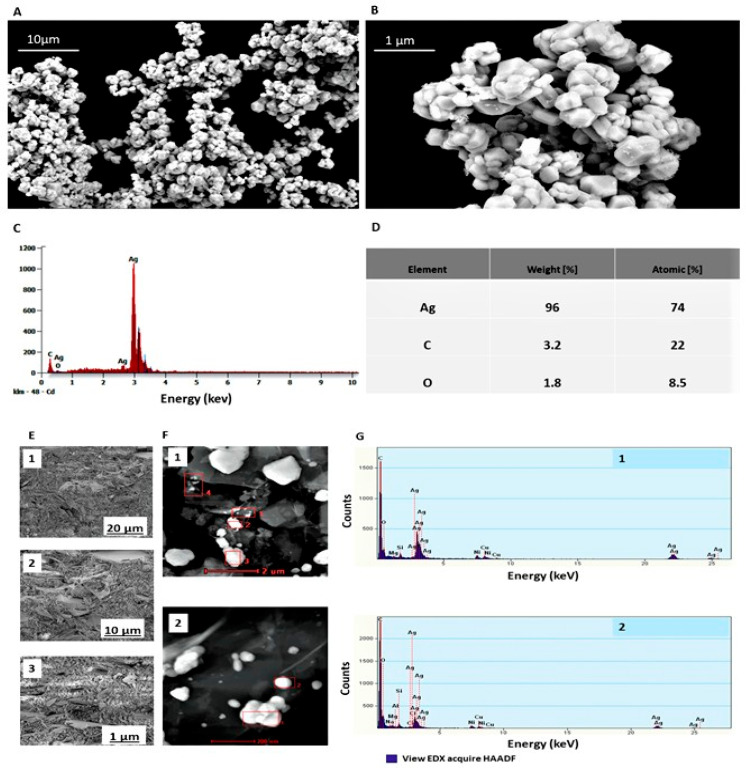
Characterisation of synthesised Ag powder utilised in this research for the formulation of Ag-Gr organic coatings (**A**–**D**). SEM images of Ag powder at EHT = 20.00 kV, WD = 15.9 mm, and signal A = SE2. 10 µm-magnification 5.00 KX (**A**); 1 µm-magnification 15.00 KX (**B**). The graph illustrates the spectrum derived from the EDS elemental composition analysis of Ag powder, with the most significant Ag signal observed at approximately 3.0 keV, thereby confirming the pure crystalline nature of the metallic Ag powder (**C**). The weight percentage of each element and the atomic composition of Ag powder are detailed (**D**). Characterisation of the developed Ag-Gr coatings (**E**–**G**). SEM images of Ag-Gr depict the structure of the cut coatings at EHT = 3.00 kV, WD = 19.4 mm, and signal A = SE2 (**E**). (1) 20 µm-magnification, 2.5 KX; (2) 10 µm-magnification, 5.00 KX; (3) 1 µm-magnification, 10.00 KX. S/TEM elemental composition analysis is presented, with red bolds highlighting four selected areas (1–4) for EDX analysis at a magnification of 2 µm (**F**). Two selected areas from area 1 at 200 nm containing Ag are shown (**G**). (1,2) The graphs (1 and 2) display the spectrum of EDX elemental composition analysis for the two selected areas from area 1 of Ag-Gr coatings.

Characterisation of synthesised Ag powder for morphology revealed spherical, agglomerated particles that formed aggregates (Figure 2A,B). Single Ag particles were difficult to identify (Figure 2B). SEM-EDS elemental composition analysis resulted in the identification of a clear Ag peak at 3.0 keV and estimated the weight percentage content of Ag (96%), C (3.2%), and O (1.8%) (Figure 2C,D).

Confirmation of the functionalization of bioactive substances (Ag, AgSiO_2_, and AgNaOPZr) and the elemental composition of coatings has been achieved using SEM and/or S/TEM, along with EDS/EDX analysis.

S/TEM and EDX elemental analysis of 5% Ag-Gr coating discs revealed areas with single and/or aggregated, spherical Ag identified at approximately 3.0 keV, which is related to surface plasmon resonance displayed by the Ag (Figure 2F). The EDX analysis of four areas indicated a clear crystalline form with the highest Ag signal at approximately 3.0 keV (area 1 and area 2), confirming the pure crystalline character of the metallic Ag for Ag-Gr organic coatings (Figure 2G(1,2)).

**Figure 3:**
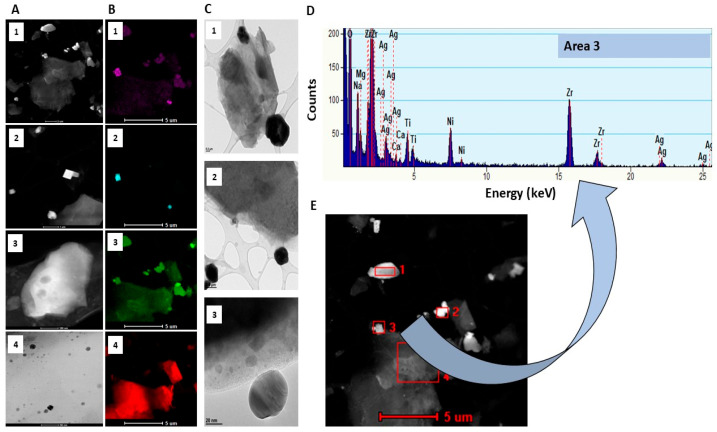
Analysis of S/TEM and EDX for the AgNaOPZr-Gr organic coatings employed in this study. S/TEM images 1–4 of AgNaOPZr-Gr particles at varying magnifications (**A**,**B**). S/TEM images 1–3 showing the shapes of nanoparticles at three different magnifications (**C**). The graph displays the spectrum of EDX elemental composition analysis for the area 3 of AgNaOPZr-Gr organic coating (**D**). EDX regions for the elemental analysis of AgNaOPZr-Gr. The analysed regions (1–4) of the organic coating AgNaOPZr-Gr particles are highlighted in red bold (**E**).

For Ag-NaOPZr-Gr organic coatings, the S/TEM characterisation of particle morphology revealed a significant diversity in sizes and shapes, including both spherical and cubical forms (Figure 3A–C). The particles were observed to be either singular or agglomerated (Figure 3D).

Furthermore, EDX analysis revealed multiple elements that constitute the matrix of the Ag-NaOPZr-Gr coatings, with the most significant peaks observed at area 3 of O (1.0 keV) and Zr (2.5 keV), in addition to the less intense peaks of Na (2.0 keV), Ag (3.0 keV), and Ti (5.0 keV) (Figure 3D). In regions 1, 2, and 4, a distinctive peak signal for Ag (3.0 keV) has not been detected (Figure 3E).

**Figure 4:**
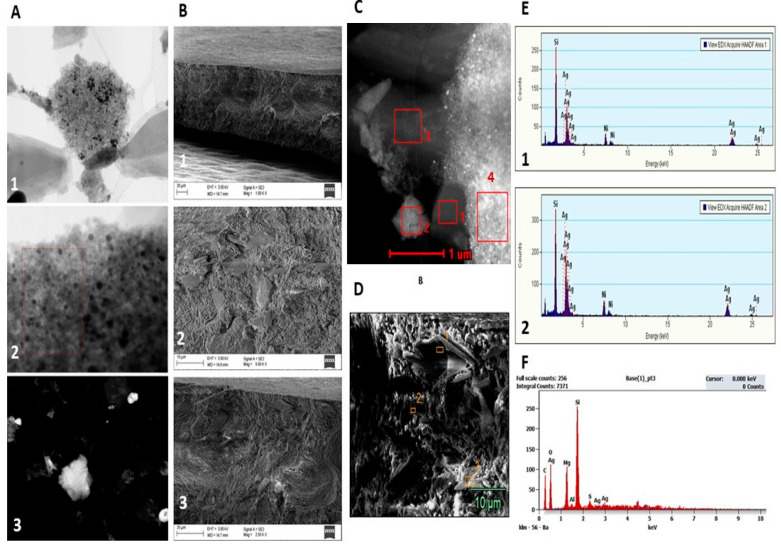
S/TEM, SEM, and EDS/EDX analysis of the fabricated AgSiO_2_-Gr coatings used in this study. S/TEM images of particles. Agglomerated particles in coatings at 500 nm (1); 100 nm (2); shape and sizes of singular particles in coatings 2 µm (3) (**A**). SEM images of AgSiO_2_-Gr coatings cross-section at 20 µm (1); 10 µm (2); 1 µm (3) (**B**). S/TEM image of analysed areas using EDX elemental composition. Analysed areas are highlighted in red bold (1–4) (**C**). SEM cross-section image of the analysed areas using EDS analysis. Analysed areas (1–3) are highlighted in orange bold (**D**). EDX of elemental composition of areas 1 and 2 (1,2) (**E**). EDS of elemental composition obtained by SEM (**F**).

The S/TEM analysis of AgSiO_2_-Gr coatings demonstrates a more uniform particle size distribution (Figure 4A(1,2)), exhibiting either a singular spherical morphology or agglomerated particles (Figure 4A(3)). The S/TEM-EDX assessment showed the most pronounced peak related to Si (2.0 keV) and a less prominent peak for Ag (3.0 keV) in Section 1 and Section 2 (Figure 4C,E(1,2)). Similarly, the SEM analysis identified the most significant peak for Si (2.0 keV), along with less pronounced peaks for the elemental composition of the Gr-coating, including C (0.2 keV), O (0.5 keV), Mg (1.0 keV), and a very weak peak for Ag (3.0 keV) (Figure 4D,F). The Ag content level (wt%) was determined to be approx. 0.6% through SEM-EDS analysis.

### 2.2. Cytotoxicity

**Figure 5:**
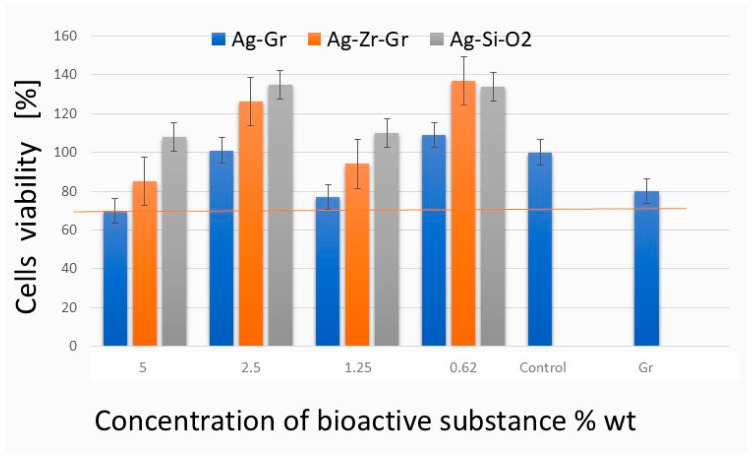
The averaged cytotoxicity results of the evaluated organic coatings were derived from three independent trials and three technical replications, utilising the NR cytotoxicity assay in accordance with the ISO 10993-5 standard [29]. The graph illustrates the percentage viability of MRC-5 cells following inoculation with the evaluated organic coatings at approximately 0, 0.625, 1.25, 2.5, and 5% of bioactive substance content. The bars represent the standard error.

The obtained results indicated that all tested coatings are non-cytotoxic to MRC-5 cells, showing cell viability of ≥70% of the control group (Figure 5). At the maximum concentration of Ag-Gr tested (5% wt), a cell death of 30% was recorded, indicating that the synthesised Ag is non-toxic and can be safely used at this concentration. For Ag-SiO_2_-Gr and Ag-NaOPZr-Gr in all concentrations, the level of viable cells exceeded 80% compared to the control (Figure 5). The results for coatings that consist of AgSiO_2_ (ca. 0.625, 1.25, 2.5, and 5%), Ag-NaOPZr-Gr (ca. 0.625 and 2.5%) and Ag (ca. 0.625%) indicated that cell viability was greater than 100% in comparison to the negative control (Figure 5).

### 2.3. Antiviral Activity Assessment of Coating Paints

#### 2.3.1. Inverted Fluorescence Microscopic Assessment of Cytopathic Effect Caused by HCoVs

**Figure 6:**
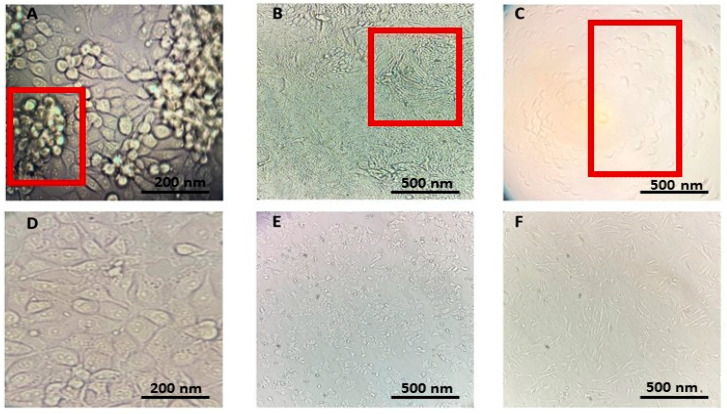
Inverted fluorescence microscopy images of the cell lines utilised for the cultivation of HCoVs-229E, OC43, and NL63. The red frames in figures (**A**–**C**) denote CPE, which refer to the observable morphological and degenerative alterations occurring 3–7 days post-infection by HCoVs (for instance, clumped cell aggregates or rounded host cells): (**A**) LLC-MK2 cell line infected with HCoV-NL63; (**B**) HCT-8 cell line infected with HCoV-OC43; (**C**) MRC-5 cell line infected with HCoV-229E. The uninfected cell lines are represented as follows: (**D**) LLC-MK2; (**E**) HCT-8; and (**F**) MRC-5.

Three HCoVs have been evaluated for cytopathic effect (CPE) formation on suitable cell lines (Figure 6A–C), and compared with uninfected cells (Figure 6D–F). In the context of the neutral red uptake (NRU) CPE assay, HCoV-229E has been identified as the model HCoV for antiviral evaluations due to its pronounced capacity to generate characteristic CPE in MRC-5 cells, with clearly visible morphological changes, including cell rounding and sloughing, appearing within a timeframe of 5–7 days post-infection (Figure 6C) in comparison to not infected cell line MRC-5 (Figure 6F).

#### 2.3.2. Evaluation of CPE Formation and NRU-CPE Assay

After 5–7 days of incubation, 96-well plates were assessed using an inverted fluorescence microscope (Figure S1). Microscopic evaluation shows non-CPE formation caused by HCoV-229E on cells incubated with HCoV-229E lysate treated with AgSiO_2_-Gr and Ag-Zr coatings in all evaluated concentrations in comparison to positive and negative control (Figure S1A,B). For Ag-Gr treatments indicating CPE formation by Ag-Gr 5% and 2.5% treated HCoV-229E lysates, similar to the positive control (HCoV-229E) not treated with coating paints (Figure S1C) (Table 2).

**Figure 7:**
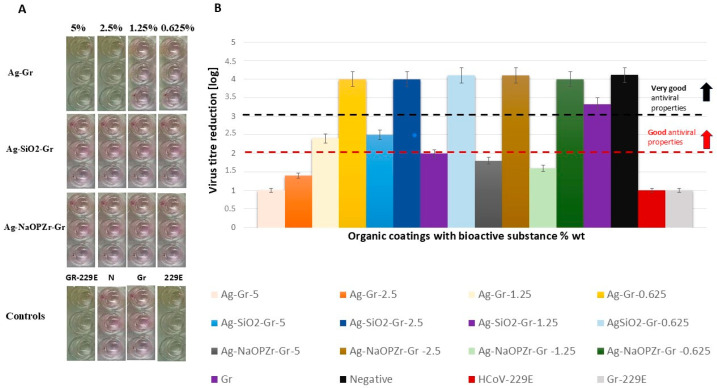
Results derived from the evaluation of antiviral activity of coatings utilising the colorimetric NRU-CPE assay. (**A**) An example of cut 96-well plates observed after a 7-day incubation period at 35 °C with 5% CO_2_; the coatings Ag-Gr, Ag-SiO_2_-Gr, and Ag-NaOPZr-Gr, which contain bioactive substances, were assessed at 5, 2.5, 1.25, and 0.625%. The blank colour of wells signifies no reduction in virus titer, while the pink colour of wells indicates a reduction in virus titer. (**B**) The graph displays the level of virus titer reduction, calculated as the average results from three independent trials and three technical replicates for each coating tested: Ag-Gr, Ag-SiO_2_-Gr, and Ag-NaOPZr-Gr at 0.625, 0.125, 2.5, and 5% wt. Lines on the graph indicate the antiviral properties’ level of assessed coatings under the ISO 21702:2019 EN standard [30]; red line indicates good antiviral properties (log ≥ 2); black line indicates very good antiviral properties (log ≥ 3) of tested organic coatings. The bars represent the standard error.

The averaged NRU-CPE assay results from replicated tests of the antiviral properties of coatings against HCoV-229E indicate the lowest reduction in the virus titer level by Ag-Gr at ca. 5% wt (log 1), and 2.5% wt (log 1.4) (Table 2) (Figure 7A,B). However, Ag-Gr coatings at ca. 1.25% and 0.625% of Ag content showed good (log = 2.4) and very good (log = 4) antiviral properties. The highest HCoV-229E reduction level was obtained by Ag-SiO_2_-treated virus lysates in all tested concentrations (0.625, 1.25, 2.5, and 5% wt) of bioactive substances (reduction compared to the negative control level) (log ≥ 2) (Table 2). The obtained results show that the infectious titer decreased by approximately 10^4^ times (RF ≅ 4 log10 TCID_50_/mL) for Ag-SiO_2_ and Ag-Zr treated lysates in ca. 0.625% and 2.5% of bioactive compound content in coatings (Table 2) (Figure 7A,B). These values corresponded to an inactivation of 99.99% of virus titer and show virucidal activity of Ag-SiO_2_ and Ag-Zr compounds. The antiviral activity of Ag-Zr coatings has not been confirmed for coatings at ca. 5% (log 1.8), and 1.25% (log 1.6) (Table 2) (Figure 7A,B).

Gr organic coatings (not containing bioactive substance) do not cause a toxic effect on cells during the incubation time (log 3.3), and do not decrease the virus titre in the time of 60 min of inoculation with the virus (log 1.1) in comparison to positive and negative control (Table 2).

## 3. Discussion

Recent research has demonstrated that AgNP serve as effective antiviral agents, successfully evaluated against various viruses both in vitro and in vivo [10,13,14,15,16]. In alignment with these findings, this study examined coatings that incorporated three distinct Ag-based bioactive compounds, namely Ag, AgSiO_2_, and AgNaOPZr, which exhibited antiviral properties at varying concentrations of the bioactive substances. Elemental analysis conducted using EDS revealed that the synthesised Ag comprised approximately 96–99% of the total weight, with minor contributions from carbon (3.2%) and oxygen (1.8%). These results affirm the high purity (96–99%) of the synthesised Ag powder, a critical factor for its antiviral effectiveness. Morphologically, the synthesised Ag powder and the evaluated coatings, Ag-Gr and AgNaOPZr-Gr, predominantly exhibited signs of particle agglomeration, as evidenced by S/TEM and SEM microscopy images, with particle sizes reaching up to 3000 nm for Ag-Gr and 1300 nm for AgNaOPZr-Gr. The results from the NRU-CPE assay indicated that Ag-Gr coatings at approximately 5% and 2.5% weight and AgNaOPZr-Gr at around 5% and 1.25% bioactive substance content did not demonstrate significant antiviral properties (log < 2). Conversely, testing Ag-Gr at lower concentrations (1.25% and 0.625%) revealed good antiviral properties (log > 2), while AgNaOPZr-Gr at approximately 2.5% and 0.625% exhibited very good antiviral properties (log > 4). In contrast, coatings containing Ag-SiO_2_ (nanoparticles sized 5–15 nm) displayed the most pronounced antiviral activity, reducing virus titres to levels comparable to negative controls, as evaluated by the NRU-CPE assay (log ≥ 2) across all four tested concentrations. Consistent with our findings, previously published studies have indicated that the size and dispersion of nanoparticles can significantly influence their antiviral efficacy, with the optimal size range being between 10 and 50 nm [10,19,31]. Smaller particles, characterised by a higher surface area-to-volume ratio, typically exhibit enhanced interactions with viral envelopes or capsids, leading to more effective inactivation [32,33].

Recent investigations into AgSiO_2_ composites reveal encouraging long-term stability when subjected to ambient conditions and mechanical stress, thereby enhancing their suitability for practical surface applications [22]. This study demonstrated that coatings containing AgSiO_2_ exhibited both good and very good antiviral properties under in vitro conditions, corroborating other studies that report favourable antibacterial and antiviral characteristics of AgSiO_2_ [21,24,34]. EDS analysis revealed that the primary component of AgSiO_2_-Gr is silicon, with a low weight percentage of Ag. The synergy of these bioactive elements (Ag and Si) may augment antiviral effectiveness, reinforcing previous conclusions that this elemental composition can surpass the efficacy of individual components in antiviral applications, exhibiting significant antiviral activity [21]. The sustained antiviral effectiveness observed in the Ag-SiO_2_ bioactive compounds (5–15 nm) across all tested concentrations can be attributed to the stabilising role of the SiO_2_ matrix, which improves the dispersion of nanoparticles and controls the release of Ag ions. This observation is consistent with prior studies suggesting that AgSiO_2_ composites maintain extended antiviral and antibacterial properties, as the SiO_2_ functions both as a physical support and a diffusion barrier for Ag ions [35,36].

Metallic nanoparticles, including AgNP, can have a cytotoxic effect on cells and accumulate in tissues such as the liver, lungs and kidneys in the human body [36]. This can lead to a higher risk of inflammatory and immunotoxicity responses in the human body. Several factors can influence the toxicity of Ag, including the form (ionic forms are considered more toxic than nanoparticle forms), exposure time to the cells, dose, NP size, shape, and type of surface coating [37,38,39,40,41,42,43,44]. In this study, we have tested the cytotoxicity of coatings using NRU assay recommended for testing the cytotoxicity of biomaterials on MRC-5 lung fibroblasts cells, with the highest level of 30% in reduction in cell viability by Ag-Gr; however, this level is still considered non-toxic, and can be related to higher concentrations (5% wt) of the Ag element in coating. Interestingly, in some samples, cell viability slightly exceeded 100% relative to the untreated control, which may indicate mild stimulation of the cellular metabolism or lysosomal activity, likely due to low concentrations of bioactive components (e.g., Ag ions or SiO_2_) released from the coatings; such effects have been reported in similar NR-based assays and are generally observed when materials are non-cytotoxic and potentially supportive of cell homeostasis [45].

This study is not comprehensive and has several limitations that must be recognised. In this study the results are limited to in vitro conditions and may not completely reflect biological responses in practical usage. While our investigation adhered to ISO 21702 by implementing a 60 min exposure duration, subsequent studies should investigate shorter exposure times to evaluate the initial kinetics of viral inactivation. Furthermore, the long-term stability of the coating, especially under conditions of mechanical wear or humidity, is a critical factor to consider. To further substantiate the practical application of these materials, additional studies should assess Ag ion leaching and antiviral durability under simulated environmental conditions. The effect of NPs size, shape, and coating application parameters on cytotoxicity has not been systematically studied in this work and remains a limitation of the current study. Future research should therefore prioritise addressing these gaps to confirm the long-term safety, efficacy, and practical application of such coatings.

## 4. Materials and Methods

### 4.1. Bioactive Substances

For the preparation of Gr organic coating, three different bioactive Ag-based compounds were used, including AgSiO_2_ (NanoSilverGuard POWDER 50K-HF) (ITP-System, Warsaw, Poland), AgNaOPZr-Gr (AlphaSan^®^ RC 2000) (Milliken, Spartanburg, NC, USA), and Ag powder produced through a chemical reduction process with Ag nitrate as the Ag precursor, and L-ascorbic acid as a reducing agent with modification to a method reported previously [46]. Briefly, Ag nitrate (Stanlab, Nakło nad Notecią, Poland) used as a precursor was dissolved in deionized water and mixed with L-Ascorbic acid (Chempur, Piekary Śląskie, Poland) at pH 6, T = 60 °C for 1 h. The obtained mixture was further centrifuged for 15 min at 3234× *g* using centrifuge 5804 (Eppendorf, Hamburg, Germany), following purification utilising a mix of demineralised water and 96% ethanol (Stanlab). The isolated product was dried at 60 °C for 10 h in a vacuum dryer (Vaucell, Malden, MA, USA) equipped with a vacuum system LVS 600 E (Welch, Waltham, MA, USA).

### 4.2. Coating Paint Preparation and Characterisation

#### 4.2.1. Preparation

Gr organic coatings for antiviral activity assessment have been prepared with the method reported previously [47] with modifications which involve the preparation of coating discs with three bioactive components: Ag, AgSiO_2_, and AgNaOPZr in four different concentrations. Each bioactive component was introduced into the liquid compositions in amounts of 0.625, 1.25, 2.5, and 5 wt%. Discs with a diameter of 20 mm were cut from dry coatings for antiviral properties testing.

#### 4.2.2. Physical and Optical Properties

Gr organic coatings were characterised for viscosity, density, gloss, hardness, colour and re-emission coefficient as reported previously [47].

#### 4.2.3. Characterisation of Synthesised Ag Powder and Gr Organic Coatings Using S/TEM, SEM and EDX

In the SEM analysis, the specimens were manually fractured to reveal fresh fracture surfaces, which were then affixed to aluminium stubs without any additional surface treatment. Observations were conducted using a SUPRA-40 (Carl Zeiss AG, Oberkochen, Germany) SEM under high vacuum conditions, focusing primarily on the cross-sections to evaluate the microstructural characteristics related to the fracture morphology. The secondary electron imaging mode was employed to deliver detailed topographical contrast. For the S/TEM analysis, Gr organic coating samples were pulverised using a mortar and pestle and diluted in 50% ethanol after being prepared on copper grids, followed by absorption and observation under high-resolution (S/TEM) TITAN 80-300 (FEI), which utilised HAADF scanning-transmission as a detection method. An energy dispersive X-ray spectrometer (EDS) was utilised for the analysis of the elemental composition of the samples.

#### 4.2.4. X-Ray Diffraction (XRD)

The qualitative phase composition of powders was carried out via XRD analysis with the diffractometer XRD 7 (Seifert-FPM, Radevormwald, Germany). Characteristic X-ray radiation of the Cu, Kα, and Ni filter was used for the investigations. Analysis was carried out in the 2θ range 10–100°. Identification of the phases was performed using Seifert and Match software (version 3.15), basing the results on the ICDD PDF-4+ catalogue from 2022.

#### 4.2.5. Electron Probe Microanalysis (EPMA)

The morphology of particles was examined using JXA-8230 electron probe microanalyser (JEOL, Tokyo, Japan). Samples were prepared by sprinkling powder onto double-sided adhesive carbon conductive tape, which was mounted on a microscopic brass holder. For each sample, electron images in secondary electron image (SEI) mode were taken—contrast depends mainly on the surface topography.

### 4.3. Cell Lines

The human lung fibroblast cell line MRC-5 (Cytion, Heidelberg, Germany) was maintained in Minimum Essential Medium (MEM) (Cytivia, VWR, Uppsala, Sweden), which was enriched with [1 g/L D-glucose, 1% non-essential amino acids (Capricorn), and 1 mM sodium pyruvate (Capricorn)], and/or EMEM (Cytion), both of which were supplemented with 10% heat-inactivated foetal bovine serum (FBS) (EurX^®^, EURx Sp. z o.o., Gdańsk, Poland), penicillin (100 U/mL), and streptomycin (100 μg/mL) (Sigma Aldrich, St. Louis, MO, USA). Additionally, human adenocarcinoma cells HCT-8 (Cytion) were cultured in Dulbecco’s Modified Eagle’s Medium Ham’s F12 (DMEM/F12) HyClone™ (Cytivia, VWR), also supplemented with 10% heat-inactivated FBS (EurX^®^), penicillin (100 U/mL), and streptomycin (100 µg/mL) (Sigma Aldrich). Renal tissue of adult rhesus monkeys LLC-MK2 (Cytion) was cultured in Medium 199, Earle’s Salts (Gibco, ThermoFisher, Waltham, MA, USA), supplemented with 2% heat-inactivated horse serum (ThermoFisher). The cell lines were grown in six-well plates (Sarstedt, Nümbrecht, Germany), 96-well microplates (Sarstedt, Nümbrecht, Germany), and 25-75 cm^2^ adherent culture flasks (Sarstedt, Nümbrecht, Germany) at a temperature of 37 °C with 5% CO_2_, before infection with HCoVs for either multiplication or antiviral testing of coating paints. Daily examinations of the cells were performed using Visiscope^®^ IT407 FL (VWR International, LLC, Radnor, PA, USA.) inverted trinocular fluorescence microscope (VWR).

### 4.4. Reference HCoV Strains and Replication on Cell Lines

For virus stock multiplication, reference strains of HCoV, specifically 229E (ATCC-VR-740) (LGC Standards, Teddington, UK), OC43 (ATCC-VR-1558) (LGC Standards), and NL63 (Małopolskie Centrum Biotechnologii, Kraków, Poland) were cultivated on suitable adherent host cells: HCoV-229E/MRC-5 (Cytion), HCoV-OC43/HCT-8 (Cytion), and NL63/LLC-MK2 (Cytion) by incubating at temperatures between 33 and 35 °C with 5% CO_2_ for a duration of 1–2 h to facilitate virus adsorption into the cells. The multiplicity of infection (MOI) was determined to be between 0.1 and 1. The adsorption process of HCoVs was halted by the addition of a neutralising agent (infection medium) that consisted of [EMEM (Cytion)/MRC-5 (Cytion), DMEM/F12 (Cytivia, VWR)/HCT-8 (Cytion), and Medium 199, Earle’s Salts (Gibco, ThermoFisher)/LLC-MK2 (Cytion) combined with 0.2–2% FBS (EurX^®^)]. Following the virus adsorption phase, 25 cm^2^ bottles were incubated at 33–35 °C and 5% CO_2_ for a period of 3–7 days, depending on the specific virus. The CPE resulting from the viral infection of the cell lines was monitored using a reverse-phase contrast fluorescence microscope AE 2000 (Motic, Wetzlar, Germany) on consecutive days of infection. After the incubation period of 3–7 days, the supernatant containing the viruses was collected, subjected to centrifugation (10,000× *g*, at temperatures between 0 and 4 °C) using ultracentrifuge 5425 (Eppendorf), and filtered through a sterile PES 25 mm, 0.22 µm syringe filter (VWR). The viral titers were quantified as 50% tissue culture infective dose (TCID50)/mL (Spearman-Karber) before conducting antiviral tests on the coating paint discs. The TCID_50_/mL of the viral supernatants obtained varied from 2.5 × 10^5^–2 × 10^6^. All work with HCoVs has been carried out at a biosafety level 2 laboratory.

### 4.5. Testing the Surface Antiviral Activity of Organic Coatings

Before performing antiviral NRU CPE assay of organic coatings, the model HCoV and cell line have been selected based on microscopy assessment of CPE formation of infected cell lines by three HCoV species in time up to seven days. The antiviral activity of organic coatings has been assessed under ISO 21702:2019 EN methodology for ‘Measurement of antiviral activity on plastics and other non-porous surfaces’ [30] with modifications as follows. Before testing antiviral activity, organic coating discs have been disinfected using a UV lamp at 254 nm under a biosafety cabinet for 1 h. The antiviral assays were conducted by introducing previously acquired virus supernatant, approximately TCID_50_/mL 2.5 × 10^5^–2 × 10^6^ (900 µL), onto each surface of the organic coating discs under examination, allowing it to remain for 60 min at a temperature of 20–22 °C. The culture medium was discarded from 96-well plates (prepared 48 h before testing), and 100 µL of HCoV supernatant that had been in contact with the organic coating discs was added in triplicate for each concentration (0%, 0.625%, 1.25%, 2.5% and 5%) after 60 min. Subsequently, 100 µL of serially diluted 1/10 (N, 10^−1^, 10^−2^, 10^−3^, 10^−4^) HCoV stock in infection medium was introduced to each well as a positive control, while 100 µL of virus-free infection medium served as a negative control. The plate was then incubated in a cell culture incubator (humidified, 5% CO_2_, T = 33 °C for HCoV-OC43, and T = 35 °C for HCoV-229E) for 1–2 h to facilitate virus adsorption. Following this incubation period, 100 µL of infection medium was added to each well of the 96-well plate, resulting in a total volume of 200 µL. The plates were subsequently incubated for a duration of 3–7 days. Daily examinations of the cells post-infection were performed using Visiscope^®^ IT407 FL inverted trinocular fluorescence microscope (VWR), documenting any alterations in cell morphology and the development of CPE. After 3-7 days of incubation, plates were tested with the method previously reported for testing antiviral compounds’ activity against HCoVs, with several modifications [48]. The NR solution, which contained [NR (Warchem, Zakręt, Poland) approximately 150 µg/mL, EMEM (Cytion)/DMEM/F12 (Cytivia), 2% FBS (EurX^®^)], was incubated for 12 h at 37 °C, followed by centrifugation (4000× *g*, 5 min, T = 20–30 °C) and filtration through a sterile PES 25 mm, 0.1 µm syringe filter (Thermo Fisher) to eliminate any NR precipitates formed. The virus infection medium was then discarded from the incubated 96-well plates, and the cells were washed by adding 200 µL of phosphate-buffered saline (PBS), pH = 7.2 (EurX^®^) containing calcium and magnesium to each well. After the washing step, 100 µL of the NR solution prepared before was introduced to each well of the plate. The plate was subsequently incubated in a cell culture incubator at 37 °C (5% CO_2_) for 2.5 h. After this time, the NR solution was removed, and the cells were washed by adding 200 µL of PBS (EurX^®^) with calcium and magnesium to each well. After washing, 100 µL of extraction solution [70% ethanol (Warchem), 10% glacial acetic acid (Warchem), and 20% distilled sterile water] was added to each well for 15 min of extraction. The plates were analysed for optical signal intensity using smartphone Spotxel^®^ Microplate Reader 2.2.2 (SICASYS Software GmbH, Germersheim, Germany) [49]. Analysis of the obtained antiviral results was performed for three technical replications of three independent trials. Normalisation was performed by comparing the optical signal values obtained with the smartphone Spotxel^®^ Microplate Reader 2.2.2. (SICASYS Software GmbH) [49] of the test samples to the control samples. The ISO 21702 virucidal efficacy testing includes three controls: a cytotoxicity control that assesses any potential harm to host cells; an untreated sample control to compare the antiviral activity with the treated sample; and a titration control to ensure that the virus stock concentration is sufficient to determine infectivity and measure the highest viral recovery rate from the sample. These controls provide key benchmarks for accurate and reliable results in virucidal testing [30]. For antiviral tests: negative control (MRC-5 lung fibroblast cells with infection medium), Gr (coating paint 0% wt. of bioactive compounds extract and HCoV-229E), positive control (cells with medium and HCoV-229E). Calibration curves of HCoV lysate dilutions as positive controls were determined for each test. Reduction factor (RF) was calculated as the logarithmic titer in the virus control minus the logarithmic titer of the treated sample with virus (RF = Δ log10 TCID_50_/mL). A log reduction of 2 or higher must be obtained in the viral particles for the material to pass the ISO 21702 virucidal efficacy testing [30].

### 4.6. Cytotoxicity Assay

The evaluation of cytotoxicity linked to organic coatings was performed utilising the NRU assay for baseline cytotoxicity, incorporating modifications to the endorsed and validated method 3T3 NRU Cytotoxicity Assay TM2007-03EU EURL ECVAM-European Union for basal cytotoxicity of chemical compounds (EURL ECVAM 2025) included as standard protocol in ISO 10993-5 Biological evaluation of medical devices—Part 5: Tests for in vitro cytotoxicity [29,50]. These modifications include the use of MRC-5 cells to evaluate the applicability of organic coatings for use in protective masks. MRC-5 cells were inoculated into 96-well cell culture plates at approximately 10^4^ cells per well and incubated for 24 to 48 h within an incubator at 37 °C, 5% CO_2_. Subsequently, the MRC-5 cells were subjected to various concentrations of bioactive compounds in organic coating extracts obtained through soaking organic coating discs in EMEM (Cytion) in time of 60 min, following incubation of 100 µL of extracts on seeded MRC-5 cells in 96-well plates for a period of 7 days. After this incubation, the culture medium was eliminated, and the cells were incubated with NR solution [NR (Warchem) approximately 150 µg/mL, EMEM (Cytion) 2% FBS (EurX) for 2.5 h. The mean signal intensity was quantified for replicates using a smartphone Spotxel^®^ Microplate Reader 2.2.2. (SICASYS Software GmbH) [38]. Viability was calculated as a percentage by comparing the optical signal intensity of untreated control cells with that of the treated cells containing bioactive organic coating disc extract. If the relative cell viability for the highest concentration of the sample extract (100% extract) is ≥70% of the control group, then the material is considered non-cytotoxic [29].

## 5. Conclusions

This research has shown that the produced Ag powder possesses a high degree of purity, thereby affirming its potential for use in antiviral applications. The Ag-Gr coatings that were developed exhibited notable antiviral activity, with a log value of 2.4, and exceptional antiviral activity, with a log value of 4, at reduced concentrations of approximately 1.25% and 0.625% of the bioactive substance content, while demonstrating no antiviral activity at concentrations around 2.5% and 5%. Furthermore, there was no indication of cytotoxicity at any of the concentrations that were tested. The broad distribution of particle sizes and the noted aggregation could restrict its antiviral effectiveness by diminishing the availability of active surface area and obstructing interactions with viral entities. These results emphasise the necessity of optimising particle dimensions and reducing agglomeration to improve efficacy. Among the nanocomposites evaluated, the AgSiO_2_-based coating exhibited the most robust and consistent antiviral activity against HCoV-229E across various concentrations, significantly surpassing the performance of Ag–Gr and AgNaOPZr–Gr formulations. Importantly, AgSiO_2_–Gr coatings achieved a log ≥ 4 reduction in viral titer at concentrations of 0.625% and 2.5%, and a log ≥ 2 reduction at 1.25% and 5%, demonstrating strong dose–response effectiveness even at relatively low silver concentrations. These findings underscore the innovative nature and potential in practical application of SiO_2_-based nanocomposites in antiviral surface treatments. However, subsequent research should focus on optimising the dispersion of nanoparticles within the coating matrix, assessing the long-term stability and potential leaching of silver, and performing ex vivo testing to confirm safety and sustained antiviral efficacy in healthcare and high-contact public environments.

## Figures and Tables

**Table 1:** Characteristics of bioactive substances used for Gr organic coatings development in this study.

Compound/Purity	Particle Size [nm]	Content of Ag [%]	Carrier/Concentration
Ag-NaOPZr/99%	up to 1300	10	N/A
Ag-SiO_2_	5–15	5	silicate/70%
Ag	up to 3000	96–99	N/A

**Table 2:** A summary of the findings derived from the evaluation of antiviral activity of Ag-Gr, AgSiO_2_-Gr, and AgNaOPZr-Gr of organic coatings inoculated with HCoV-229E, conducted in three replicates and three independent trials over time, utilising the NRU-CPE assay and analyses for CPE-formation on MRC-5 cells through an inverted fluorescence microscope.

Name	ca. [%] ^1^	CPE ^9^	RF [log]
Ag-Gr ^2^	5.0	(3)	1.0
	2.5	(3)	1.4
	1.3	(0)	2.4
	0.6	(0)	4.0
Ag-SiO_2_-Gr ^3^	5.0	(0)	2.5
	2.5	(0)	4.0
	1.3	(0)	2.0
	0.6	(0)	4.1
Ag-NaOPZr-Gr ^4^	5.0	(2)	1.8
	2.5	(0)	4.1
	1.3	(2)	1.6
	0.6	(0)	4.0
Control-Gr ^5^	0.0	(0)	3.3
Control-Gr-229E ^6^	0.0	(3)	1.1
Control-N ^7^	0.0	(0)	4.1
Control-229E ^8^	0.0	(3)	1.1

^1^ Concentration of bioactive substance; ^2^ Gr organic coatings with Ag; ^3^ Gr organic coatings with AgSiO_2_; ^4^ Gr organic coatings with AgNaOPZr; ^5^ Gr organic coatings 0% of bioactive substance; ^6^ Gr organic coatings with 0% of bioactive substance inoculated with HCoV-229E; ^7^ Cells with infection medium; ^8^ Cells with HCoV-229E; ^9^ CPE caused by HCoV-229E on MRC-5 measures as percentage of affected cells in the well: (0) 0–29%; (1) 30–59%; (2) 60–80%; (3) >80%.

## Data Availability

The original contributions presented in this study are included in the article/Supplementary Material. Further inquiries can be directed to the corresponding authors.

## Supplementary Materials

The following supporting information can be downloaded at https://www.mdpi.com/article/10.3390/ijms262211068/s1.
