# Supplementary material for: Features and In Vitro Assessment of Antiviral Activity of Organic Coatings Doped with Silver-Based Compounds Against Human Coronavirus
Source: Int J Mol Sci. 2025 Nov 15;26(22):11068. doi: 10.3390/ijms262211068 (PMC12652288; doi:10.3390/ijms262211068)
Supplement: Supplementary file 1 [file ijms-26-11068-s001.zip › ijms-3898084-supplementary.pdf]

Table S1. Particle size distribution of the produced silver powder.

Powder	Particle size distribution, μm		
Ag	D10	D50	D90
	0.070	2.621	6.484

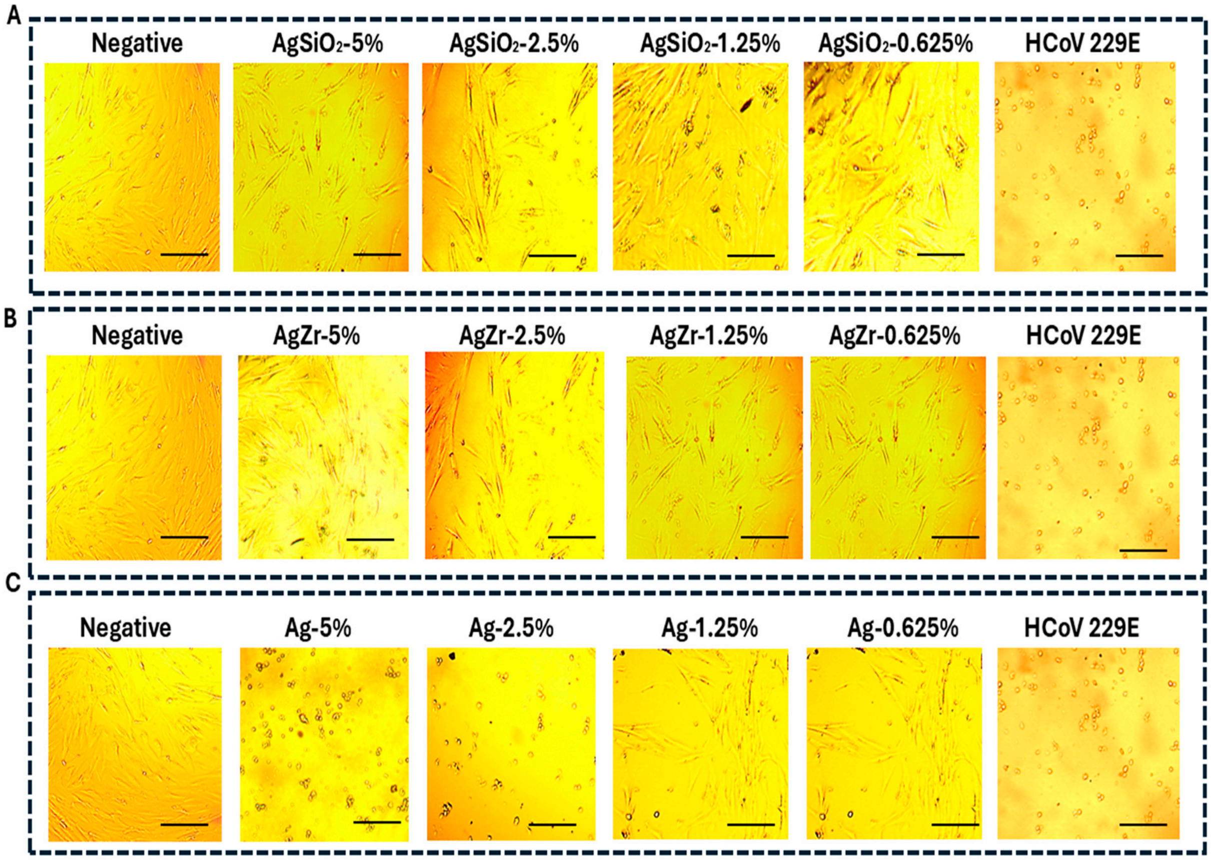

Figure S1. CPE caused by HCoV-229E on lung fibroblast cells MRC-5 treated with developed coating paints (*ca.* 5%, 2.5%, 1.25%, and O.625%) for 1h, negative control (EMEM, and 2% FBS), and positive control (HCoV-229E) after 5-7 days of incubation at 35°C, 5% CO_2. (**A**) MRC-5 incubated with 100 μl of suspension HCoV-229E/Ag-SiO_2 coating paints in range of concentrations (**B**), MRC-5 incubated with 100 μl of suspension HCoV-229E/Ag-Zr coating paints in a range of concentrations (**C**), and MRC-5 incubated with 100 ul of suspension HCoV-229E/Ag coating paints in a range of concentrations.
